# Dependence of viscosity of suspensions of ceramic nanopowders in ethyl alcohol on concentration and temperature

**DOI:** 10.1186/1556-276X-7-412

**Published:** 2012-07-23

**Authors:** Gaweł Żyła, Marian Cholewa, Adam Witek

**Affiliations:** 1 Department of Physics, Rzeszów University of Technology, Aleja Powstańców Warszawy 6, Rzeszów, 35-905, Poland; 2 Institute of Ceramics and Building Materials, Warsaw, 02-676, Poland

**Keywords:** Viscosity dependence, Ceramic nanopowders, Yttrium oxide, YAG, Magnesium aluminum spinel

## Abstract

This work presents results of measurements of viscosity of suspensions including yttrium oxide (Y_2_O_3_), yttrium aluminum garnet (Y_3_Al_5_O_12_) and magnesium aluminum spinel (MgAl_2_O_4_) nanopowders in ethanol. Nanoparticles used in our research were either commercially available (Baikowski) or nanopowders newly developed in the Institute of Ceramics and Building Materials in Warsaw, Poland. The study was conducted in a wide range of shear rates (0.01 to 2,000 s^−1^) and temperature interval from -15°C to 20°C. A Haake Mars 2 rheometer from Thermo Fisher, Germany, was used in the Biophysics Laboratory at Rzeszów University of Technology. Most of the samples show a non-Newtonian behaviour. It was confirmed with a Rheo-NMR system from Bruker that 10% by weight of Y_2_O_3_ suspension is a non-Newtonian fluid. In this work, we also report an unexpected behaviour of the viscosity of some samples (Y_2_O_3_ and Y_3_Al_5_O_12_) due to sedimentation effect.

## Background

An interesting research has been been carried out worldwide on the characteristics of nanofluids and their possible technological applications. Nanoparticle suspensions have been of great interest to science and industry which have been described by many authors. The thermal conductivity of some of these materials is higher than the thermal conductivity of pure liquid [1]. Li et al. described a two-step method for producing nanofluids by distributing basic nanopowders in liquid [2]. Since the discovery, there have been numerous studies performed on the thermal conductivity of nanofluids [3-8]. Thermal and rheological properties of nanofluids have been described by many authors, including Chen and Ding [9], Prasher et al. [10], Pastoriza-Gallego et al. [11,12] and He et al. [13]. There are also many reports describing the rheological properties of suspensions of nanopowders [14,15] and nanofluids [16-18]. Heine et al. [15] noted that the rheological properties of nanopowder suspensions are affected by the shape of nanoparticles. The problem on the shape of the nanoparticles has been also analyzed by Chen et al. [19]. There are many exciting possibilities of applications of nanofluids [20-23]. Saidur et al. [24] have written more on their potential applications.

All nanomaterials investigated in this work can be used to produce high-quality advanced ceramics [25-28]. The yttrium oxide (Y_2_O_3_) powder is used as a raw material for single-crystal growth and for yttria ceramic material sintering. Since birefringence does not apply to cubic ceramics and their transparent forms are possible, various optical applications of yttria have been reported [29-31]. Due to the high-temperature melting point of yttrium oxide, crystal growth by the Czochralski method is cumbersome and yttria sintering seems to be the solution for bulk transparent media manufacturing. On the other hand, rheological properties of yttrium oxide powder and its microstructure, negligible for the crystal growth, from the standpoints of the sintering subroutines are essential. The real ceramic transparency needs the elimination of the last hundredths of percent of porosity. As a consequence of the extreme difficulty of eliminating it, most approaches to transparent ceramics use high-temperature sintering which is associated with a drawback of extended grain growth. To reduce this parasitical growth, the nanopowder is used as a starting material. This is especially important for the high-temperature-sintered ceramics like yttria. Another demand for the nanostructured yttrium oxide comes from its application as a sintering additive. The yttrium oxide is a ‘working horse’ as an additive for sintering several non-oxide ceramics. For example, very-hard-to-sinter aluminum nitride could be formed as a ceramic with at least 0.5 mol% of Y_2_O_3_. On the other hand, oxygen impurities introduced via additives strongly influence the material properties of non-oxide ceramics. One can expect that the nanostructured additives could do the job on a reduced content level. Taking into account the aforementioned application of yttrium oxide, knowledge on the rheological properties of Y_2_O_3_ nanopowder suspensions is desperately needed. The composition 3Y_2_O_3_:Al_2_O_3_, so-called yttrium aluminum garnet (Y_3_Al_5_O_12_, YAG), adopts a cubic structure and is an important material for solid-state laser rods for infrared lasers (*λ *= 1,062 nm) when doped with neodymium (Nd:YAG). The laser rods in the form of high-quality Nd:YAG single crystals fabricated by the Czochralski method are widely used. It is important to point out that the Nd:YAG single crystal, which is commonly produced by the Czochralski method, has a number of limitations. The Nd concentration that affects its performance in laser applications is limited to 1.4 at.% as a result of the segregation and distribution coefficients. Effective laser oscillation performed successfully for the first time by Ikesue [32] using Nd:YAG ceramics turned the attention to transparent YAG ceramics. Heavily doped (up to the 5 at.%) transparent Nd ceramics can be produced by starting with phase-pure YAG nanopowders or by reactive sintering approach in which a mixture of metal oxides is sintered [33]. Up to date, the most powerful solid-state heat-capacity laser has been built on the basis of ceramic Nd:YAG slabs prepared through slip casting method by Konoshima. Further development of this very promising slip casting technology requires more investigations on the rheology of YAG nanopowder suspensions. Magnesium aluminum spinel (MgAl_2_O_4_) has long been considered an attractive material for high-temperature structural applications because of its low density (3.57 g cm^−3^) and high melting point (2,135°C). Thanks to their cubic symmetry and flexural strength, spinel ceramics recently attract big interest since there is an increasing need in the military sector for high-strength, robust materials which have the capability to transmit light around the visible and mid-infrared (1 to 5 *μ*m) regions of the spectrum. The spinel ceramic is the material fulfilling the aforementioned demands and is supposed to be used as base for transparent armors and ‘heat-seeker’ missile domes. The current materials of choice for high-speed infrared-guided missile domes are single-crystal sapphire and AlON ceramics. Unfortunately, the optical transmission of sapphire and AlON does not extend to cover the entire mid-infrared range. The optical transmission of sapphire and AlON starts to drop off at wavelengths greater than approximately 4.5 *μ*m even at room temperature. The birefringence of single-crystal sapphire domes is negative as well, so the solution for precision aiming of high-speed infrared-guided missiles requires ceramic spinel domes. The spinel ceramics overcome traditional compromise between optical bandgaps and mechanical durability of domes. The transparent spinel ceramics could be obtained via reactive sintering of oxides or the synthesized spinel powder could be pre-sintered to the form of ceramic with closed porosity and hot isostatically pressed to densify to the transparent form [34]. In the second method, the starting material is the nanopowder of spinel, and their rheology is crucial for green body preparation and as a consequence of the sintering and post-sintering HIP process.

## Methods

The materials included in our investigation were suspensions of ceramic nanopowders such as (a) yttrium oxide (Y_2_O_3_), (b) YAG (Y_3_Al_5_O_12_) and (c) magnesium aluminum spinel (MgAl_2_O_4_) in ethanol. Samples were prepared at a concentration up to 20% in mass fraction. The average size of the nanoparticles measured by XRD was 31 ± 1 nm for Y_2_O_3_ nanoparticles, 40 ± 1 nm for MgAl_2_O_4_ and over 100 nm for Y_3_Al_5_O_12_.

### Sample preparation

**Figure 1:**
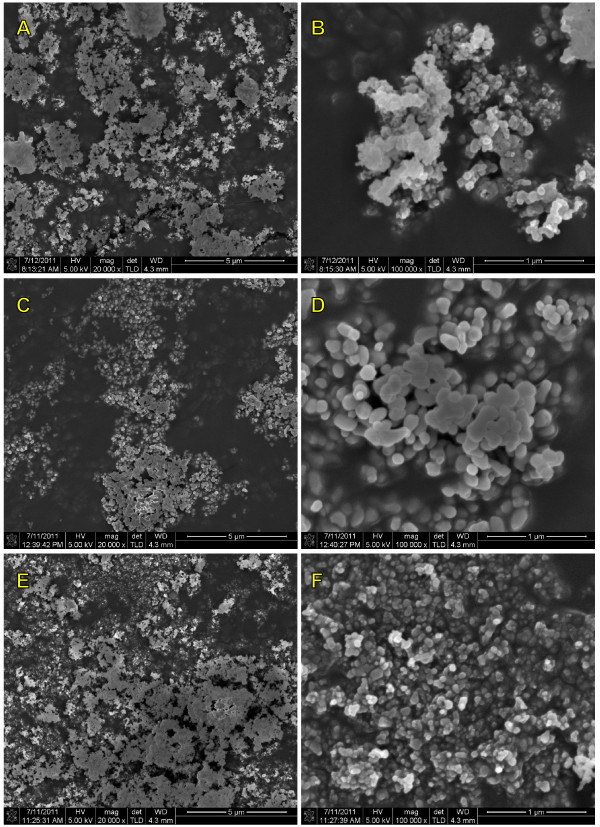
**SEM images of used nanopowders.** (**A**, **B**) Y_2_O_3_, (**C**, **D**) Y_3_Al_5_O_12_, (**E**, **F**) MgAl_2_O_4_.

The tests were performed on samples containing different concentrations of nanopowders in ethyl alcohol. Images taken with a scanning electron microscope (SEM) clearly showed the tendency of these nanomaterials to create larger conglomerates. Images taken with SEM are presented in Figure 1.

Samples were prepared on a RADWAG AS 220/X analytical balance (Radom, Poland) with an accuracy of 0.1 mg. The first phase of preparing the material for the study involved placing the nanopowder in a glass vessel and accurately measuring the required concentration. Then, the vessel was filled with an appropriate amount of ethyl alcohol (96% pure p.a., CAS: 64–17–5, POCH, Gliwice, Poland). The sample was subjected to mechanical stirring for 30 min and then placed in an ultrasound wave bath (Ultron U-505, Olsztyn, Poland) for 60 min. In [35], Ghadimi et al. presented a table which reviewed properties of various nanofluids produced during ultrasound process. They displayed the duration of action of the ultrasound in the case of different fluids. These times start from 15 min; we have used a time of 60 min for sonification after previous mechanical stirring. Subjecting the material to the ultrasound resulted in the breakdown of nanopowder agglomerates [2,35,36]. All materials used in our rheological measurement are described in Table 1. The ultrasonic cleaner, which was used to break up agglomerates, has only sample warm-up options up to a temperature of 55°C. However, an elevated temperature might help break conglomerates but at the same time causes the alcohol to evaporate quickly. It evaporates fast even at room temperature, and this process will change the real concentration of nanoparticles in the sample. Ghadimi et al. [35] describe briefly the process of evaluating the sedimentation process in nanofluids on the basis of optical pictures taken sequentially outside the rheometer. We used the same technique in observing the sedimentation process during our experiments. Unfortunately, this is only a qualitative method.

**Table 1:** Characteristics of materials used in the experiments

**Nanoparticle**	**Base fluid**	**Stability process**	**Sedimentation**
Y_2_O_3_	Ethyl alcohol	Ultrasonic cleaner	Minutes after preparation
Y_3_Al_5_O_12_	Ethyl alcohol	Ultrasonic cleaner	Hours after preparation
MgAl_2_O_4_	Ethyl alcohol	Ultrasonic cleaner	Days after preparation

### Measurement procedure

Studies of the viscosity of the nanopowder suspensions were made using a Haake Mars 2 rheometer (Thermo Electron Corporation, Karlsruhe, Germany). The geometry used during experiments was a double cone (diameter 60 mm, cone angle 1°) with a titanium layer. All measurements were conducted at a constant gap of 0.054 mm. A Peltier system and thermostat (Phoenix 2, Thermo Electron Corporation) was used to control the temperature. In addition, a measuring system was isolated from the environment using glass rings. We performed measurements of dynamic viscosity in a shear rate range of 0.01 to 2,000 s^−1^ and a temperature range of -15°C to 20°C. In our experiments, we made every effort to reduce the sedimentation process by placing the sample in the rheometer in less than 100 s after preparing it in the ultrasonic bath.

## Results

**Figure 2:**
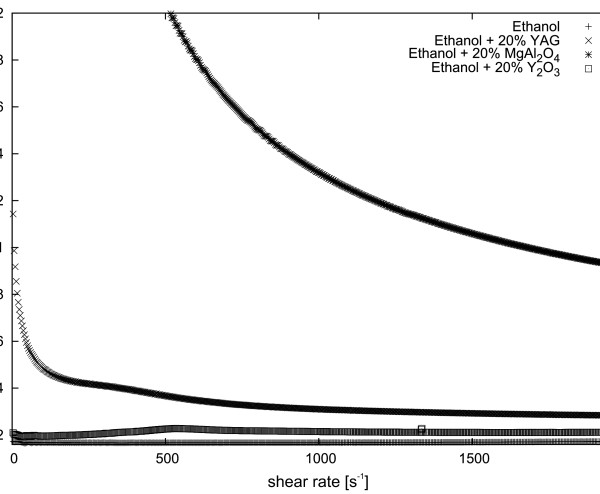
**Dynamic viscosity dependence of 20 wt. % Y**_**2**_**O**_**3**_**, Y**_**3**_**Al**_**5**_**O**_**12**_**, MgAl**_**2**_**O**_**4**_**suspensions in ethyl alcohol at 10°C.**

**Figure 3:**
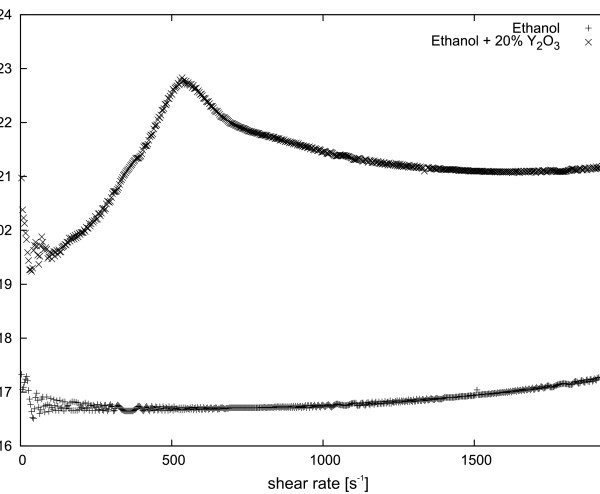
**Summary of dynamic viscosity of pure ethyl alcohol and 20 wt. % Y**_**2**_**O**_**3**_**suspension at 10°C.**

**Figure 4:**
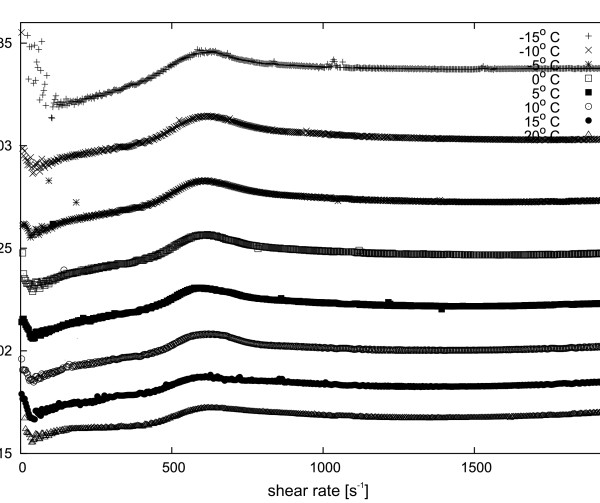
**Dynamic viscosity dependence of 15 wt. % Y**_**2**_**O**_**3**_**nanopowder suspension in ethyl alcohol at various temperatures.**

**Figure 5:**
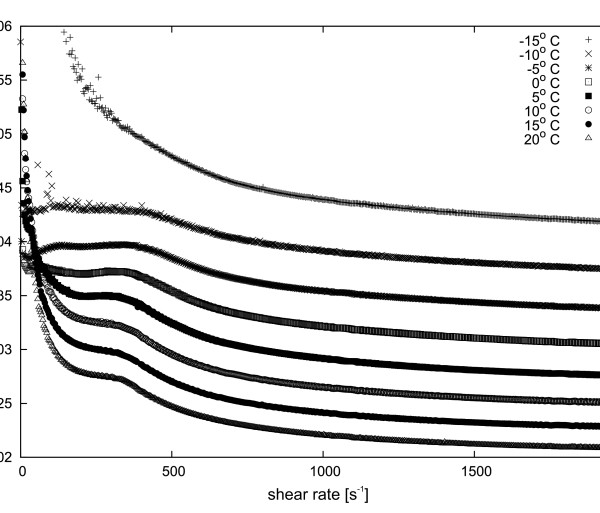
**Dynamic viscosity dependence of 15 wt. % Y**_**3**_**Al**_**5**_**O**_**12**_**nanopowder suspension in ethyl alcohol at various temperatures.**

**Figure 6:**
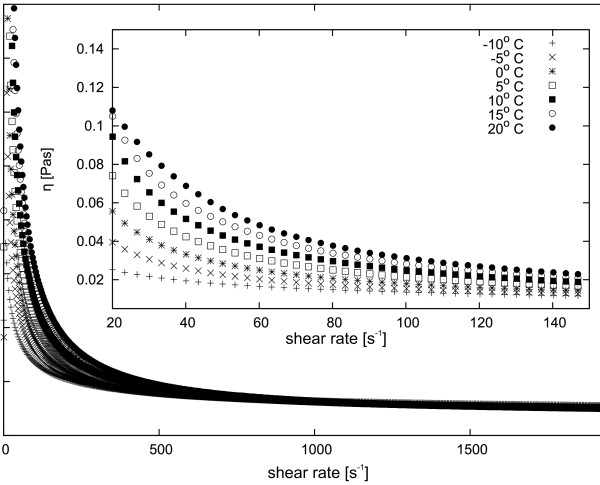
**Dynamic viscosity dependence of 15 wt.% MgAl**_**2**_**O**_**4**_**nanopowder suspension in ethyl alcohol at various temperatures.** Insert shows a close-up of the main plot.

**Figure 7:**
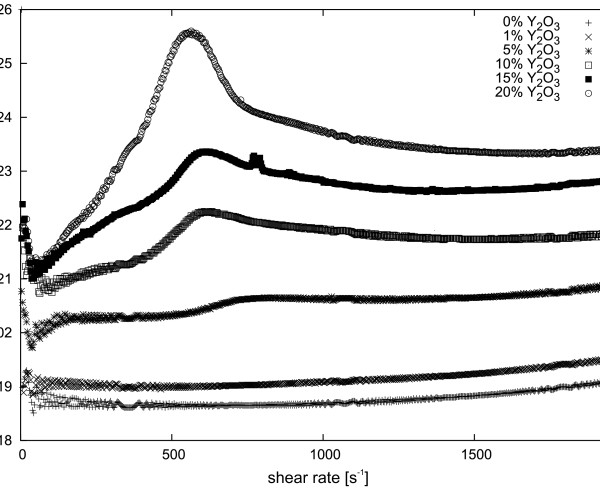
**Dynamic viscosity of various weight concentrations of Y**_**2**_**O**_**3**_**nanopowder suspensions in ethyl alcohol at 5°C.**

**Figure 8:**
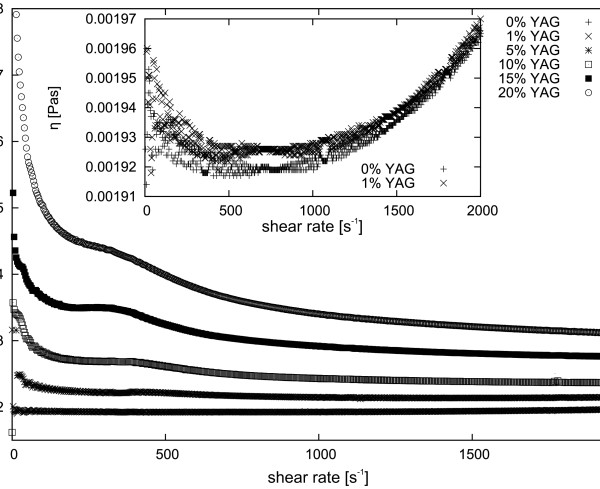
**Dynamic viscosity of various weight concentrations of Y**_**3**_**Al**_**5**_**O**_**12**_**nanopowder suspensions in ethyl alcohol at 5°C.** Insert shows a close-up of the main plot.

**Figure 9:**
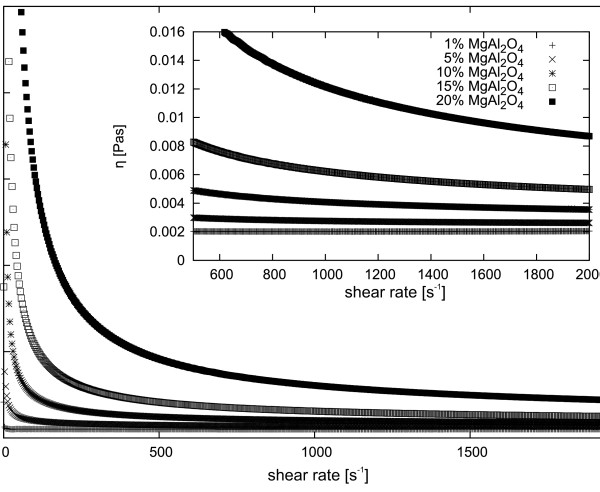
**Dynamic viscosity of various weight concentrations of MgAl**_**2**_**O**_**4**_**nanopowder suspensions in ethyl alcohol at 5°C.** Insert shows a close-up of the main plot.

In our study, the dynamic viscosity of ceramic nanopowder suspensions was investigated. Figure 2 presents the result of the measurement of dynamic viscosity of 20 wt.% nanoparticle suspensions in ethyl alcohol at 10°C for all three nanomaterials. Figure 3 shows the viscosity of only 20 wt.% of Y_2_O_3_ together with the viscosity of the alcohol. Furthermore, in Figure 4, the dynamic viscosity of the Y_2_O_3_ nanopowder suspension in ethyl alcohol for various temperatures is presented. In Figure 5, the dependence of the dynamic viscosity of the Y_3_Al_5_O_12_ nanopowder suspension in ethyl alcohol at temperatures from -15°C to 20°C is shown. In Figure 6, results of dynamic viscosity measurement of the suspension with 15 wt.% concentration of MgAl_2_O_4_ in ethyl alcohol at different temperatures is presented. In Figure 7, results of the investigation of dynamic viscosity for various concentrations of the Y_2_O_3_ nanopowder suspended in ethyl alcohol at 5°C is shown. Furthermore, Figure 8 summarizes the results of measurements of dynamic viscosity of the suspension of various concentrations of Y_3_Al_5_O_12_ in ethyl alcohol at 5°C. Finally, Figure 9 shows the dependence of dynamic viscosity on the concentration of the MgAl_2_O_4_ nanopowder in ethyl alcohol at 5°C.

**Figure 10:**
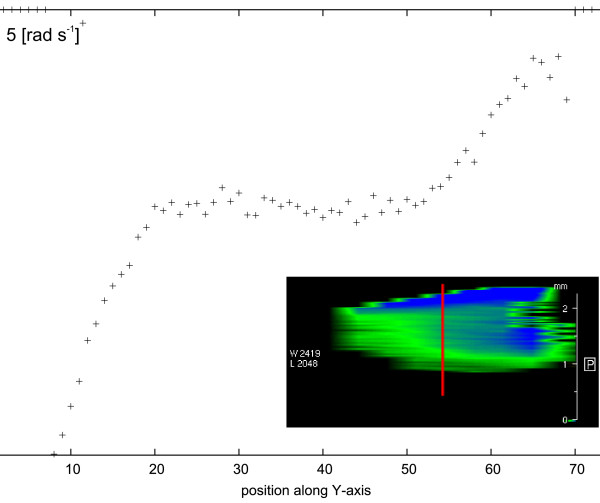
**Rheo-NMR measurement showing velocity distribution in 10 wt. % Y**_**2**_**O**_**3**_**in ethyl alcohol at 5.0-rads**^**−1**^**rotor speed.**

Measurements made at the Rzeszów University of Technology were further confirmed in the Bruker BioSpin Laboratory in Germany. For this part of research, the measuring system consisted of AV 300 WB III and was equipped with a Rheo-NMR module [37,38]. Measurements were performed in a plate-cone (diameter 16 mm, cone angle 7°) measuring geometry. We performed two series of measurements at speeds of 2.5 and 5.0 rad s^−1^ on a 10 wt.% concentration of Y_2_O_3_ nanopowder. Results of the investigation with Rheo-NMR are shown in Figure 10 which shows the velocity distribution inside the sample, depending on the distance from the stationary plate.

## Discussion

It could be seen from Figure 2 that the viscosity of the investigated materials is varied. In Figure 3, the dynamic viscosity of 20 wt.% Y_2_O_3_ nanoparticle suspensions in ethyl alcohol at 10°C is presented. With experiments performed on RheoScope in Thermo Fisher Laboratories in Karlsruhe, Germany, it could be concluded that an increase of viscosity in small shear rates, as is shown in Figure 3, had resulted in sedimentation.

The nanoparticles of Y_2_O_3_ fall at the bottom of the used measuring geometry, but only below a critical shear rate (below $\dot{\gamma }=535\;{\text{s}}^{-1}$ for this measurement). In the case of Y_2_O_3_ at $\dot{\gamma }=535\;{\text{s}}^{-1}$, the nanopowder is ‘picked up’, and nanoparticles are distributed in the whole volume of the sample. Figure 4 shows dependence of the dynamic viscosity of the 15 wt.% Y_2_O_3_ suspension in ethanol on various temperatures. The dynamic viscosity clearly decreases with increasing temperature. Similarly, in the case of Y_3_Al_5_O_12_, as shown on Figure 5, there is also an increase in temperature which causes a decrease of dynamic viscosity. In our opinion, in the case of the Y_3_Al_5_O_12_ suspension, due to the sedimentation process, a novel behaviour of the viscosity as a function of the shear rate has been observed. At a temperature of -15°C, the base fluid viscosity is so large that no sedimentation of nanopowder has been observed. However, with increasing temperature, the viscosity of the fluid decreases and nanoparticles fall at the bottom of the geometry due to the gravitational field. In the case of YAG, sedimentation is not as immediate as in the case of Y_2_O_3_, so the viscosity curves of these two suspensions are not analogous. Figure 6 shows the dependence of dynamic viscosity of the MgAl_2_O_4_ suspension as a function of shear rate at various temperatures. Unlike the two previous suspensions, the MgAl_2_O_4_ suspension is characterized by a slight decrease of viscosity with increasing temperature. In addition, sedimentation in the sample is very slow. The nanopowder concentration in the suspension also has a very significant impact on its viscosity. We investigated samples that were prepared and described with a concentration by weight. A Y_2_O_3_ nanopowder suspension shows a marked increase in viscosity with increasing concentration, as shown in Figure 7. In the case of the Y_3_Al_5_O_12_ suspension, the increase in viscosity is also clearly visible in Figure 8. Figure 9 presents dynamic viscosity of various concentrations of MgAl_2_O_4_ nanopowder in alcohol. There is a clear trend of increased viscosity with increasing concentration of nanoparticles in the suspension. Additionally, a visible change can be seen in the nature of Newtonian fluids at low concentrations and non-Newtonian behaviour at concentrations above 10 wt.%. Rheo-NMR measurements showed the non-Newtonian nature of the suspension of 10 wt.% Y_2_O_3_ in ethyl alcohol. In the case of the Newtonian fluid, the velocity distribution between the stationary plate and rotating rotor is linear. As can be seen in Figure 10, the velocity distribution in the tested sample is non-linear. The graph shows the velocity distribution in the sample at the place marked on the image by a red line. At 2.5 rad s^−1^, the velocity distribution is linear.

## Conclusions

In this work, the experimental data of viscosity measurements of suspensions of three different ceramic nanopowders have been presented. The systematic experiments have been carried out and show that the temperature and concentration of nanopowders in the base fluid has a significant influence on the viscosity of suspensions. The addition of ceramic nanopowders to the fluid (in our case, ethyl alcohol) will change its character from being Newtonian to non-Newtonian in most measured cases. In addition, apart from that, we showed that sedimentation in suspensions of such materials has an impact on the results of viscosity measurements. To prevent sedimentation of nanoparticles, the application of shear rates above the critical value is necessary. In the case of MgAl_2_O_4_ suspensions in ethyl alcohol, it showed that temperature does not significantly affect viscosity. However, the change of concentration has a significant influence on the behaviour of this sample and leads to an increase of viscosity and modifies the material from being Newtonian to a non-Newtonian one. We are currently planning further measurements to accurately determine the impact of sedimentation on the rheological properties of nanosuspensions. We also established necessary contacts to access RheoScope facilities which we do not have in our laboratory to investigate the effect of sedimentation in greater detail in the future.

## Abbreviations

SEM: Scanning electron microscope; YAG: Yttrium aluminum garnet (Y_3_Al_5_O_12_).

## Competing interests

The authors declare that they have no competing interests.

## Authors’ contributions

GŻ performed the sample preparation, characterization and experimental measurements and participated in the critical discussion of the results. MC coordinated the research project and participated in the critical discussion of the results. AW prepared the materials for research. All authors read and approved the final manuscript.

## Authors’ information

GŻ is an MSc and an assistant and PhD student at Rzeszów University of Technology. MC is an associate professor at Rzeszów University of Technology and responsible for all research activities in the Physics Department. AW is an associate professor and the Director of the Department of Nanotechnology at the Institute of Ceramics and Building Materials.
